# Usage behavior and health benefit perception of youth in urban parks: A case study from Qingdao, China

**DOI:** 10.3389/fpubh.2022.923671

**Published:** 2022-08-03

**Authors:** Wenfei Yao, Jiayue Yun, Yiping Zhang, Tian Meng, Zhiyue Mu

**Affiliations:** Department of Landscape Architecture, College of Architecture and Urban Planning, Qingdao University of Technology, Qingdao, China

**Keywords:** urban park, health perception, usage behavior, public health, youth

## Abstract

With the development of the urban park, people's cognition of the relationship between the environment and public health has been improved, and higher requirements for the living environment have been put forward. As an important group of park users, youths often have different needs regarding the health benefits brought by urban parks. To explore the potential relationship among youth's satisfaction with urban parks, their usage behavior, and health benefit perception, this article presents some constructive suggestions for the development of healthy landscapes in urban parks. Researchers have selected five typical urban parks from different areas in Qingdao, China. Furthermore, we have used the survey method by combining the practices of “issuing questionnaires, observing and interviewing” with the goal of collecting data on 500 park visitors in autumn, including information on social demography, the satisfaction of park landscape variables, and usage behavior and health perception. A linear regression model has been used to analyze the correlation among “landscape variables,” “usage behavior,” and “health benefit perception.” Results have shown that urban green landscapes and waterscapes can significantly affect youth's social health perception and static behavior. Moreover, static behaviors such as relaxation have a great impact on mental health perception. The results of this study will be beneficial in understanding youth's needs for landscapes when using urban parks. In addition, it will provide insight for the urban planners and landscape designers to design urban parks from the perspective of youth.

## Introduction

With the rapid development of the social economy and the improvement of people's living standards, “health” has become a main focus of attention. From the “Lungs of London,” “Emerald Necklace Park in Boston,” and “Central Park in New York” in the 18th century to the concept of a “Healthy City” proposed in recent years (1), all of these projects are closely related to the issue of public health. Urban parks can not only regulate the microclimate (2, 3), reduce air pollutants (4), but they can also be beneficial to physical and mental health (5), as they can reduce mental fatigue (6), relieve pressure (7), effectively improve cognitive ability (8), increase vitality (9), provide opportunities to participate in public activities, and promote public health benefits (10). The range of this research has been extended to include medical landscapes (11), psychological and social health (12), and all kinds of urban health benefits (13, 14). The whole process is concerned with the needs of people and reflects the significance and value of urban park construction. In addition, existing studies show that human behavior is affected by various factors (15, 16). Rachel and Stephen (17) believe that all human thoughts and behaviors are carried out in numerous physical environments with unique characteristics. The most important connection between the environment and the mind includes the quality and well-being of certain environments (most obviously the natural environments), and people can gain different green space experiences in different environmental characteristics. Plant resources have special environmental, scientific, cultural, aesthetic, and recreational importance (18). Waterscape has a high-aesthetic quality, entertainment, and ecological protection value. Streets and plazas encourage walking and open spaces with high gathering potential (19). Therefore, the type and quality of activity space have a great influence on people's activities (20, 21). However, in order to reduce the deviation caused by different types of urban parks, the same types of urban park spaces such as waterscapes, squares, or hillside fields have been selected for measurement in this study.

Different groups of people have different usage demands and health benefit perceptions of urban parks (22). For example, people under stress prefer to stay in natural environments with rich vegetation (23), socially vulnerable groups need more secure and barrier-free infrastructures (24), and college students prefer to have a good visual environment in urban parks during the COVID-19 pandemic (23). Therefore, how to construct an urban park landscape that can meet the needs of different groups has become a critical issue. Urban parks are a useful environmental source for improving the physical, mental, and social health of urban residents. Yet, recent studies have shown that parks are generally underutilized among youths (25). Youth often experience frequent mental and psychological health problems. Their highly variable personal characteristics, make them a special group to be concerned about (26). China is a developing country with an aging population. There is a gap between China and the international community in the terms of population age group. Therefore, this study is based on the definition of age groups in China (27). According to the 2019 population, sampling survey conducted by the National Bureau of Statistics of China (28), youth (aged 15–35 years) account for about 32% of the population, which was a significant proportion of the total population. To explore the potential relationship among park landscape variables, youth's usage behavior, and health benefit perception, and to put forward suggestions on the construction of an urban park health landscape. At first, we examined the influence of different spatial types on urban park use behaviors. Second, we aimed to discover which landscape variables had a significant impact on usage behavior and perceived health. At last, we explored the relationship between usage behavior and health perception. The results of this study help us to better understand the factors affecting the use of parks by youth, effectively improve their willingness to use parks and enhance their health as well.

## Materials and methods

### Study area

**Figure 1:**
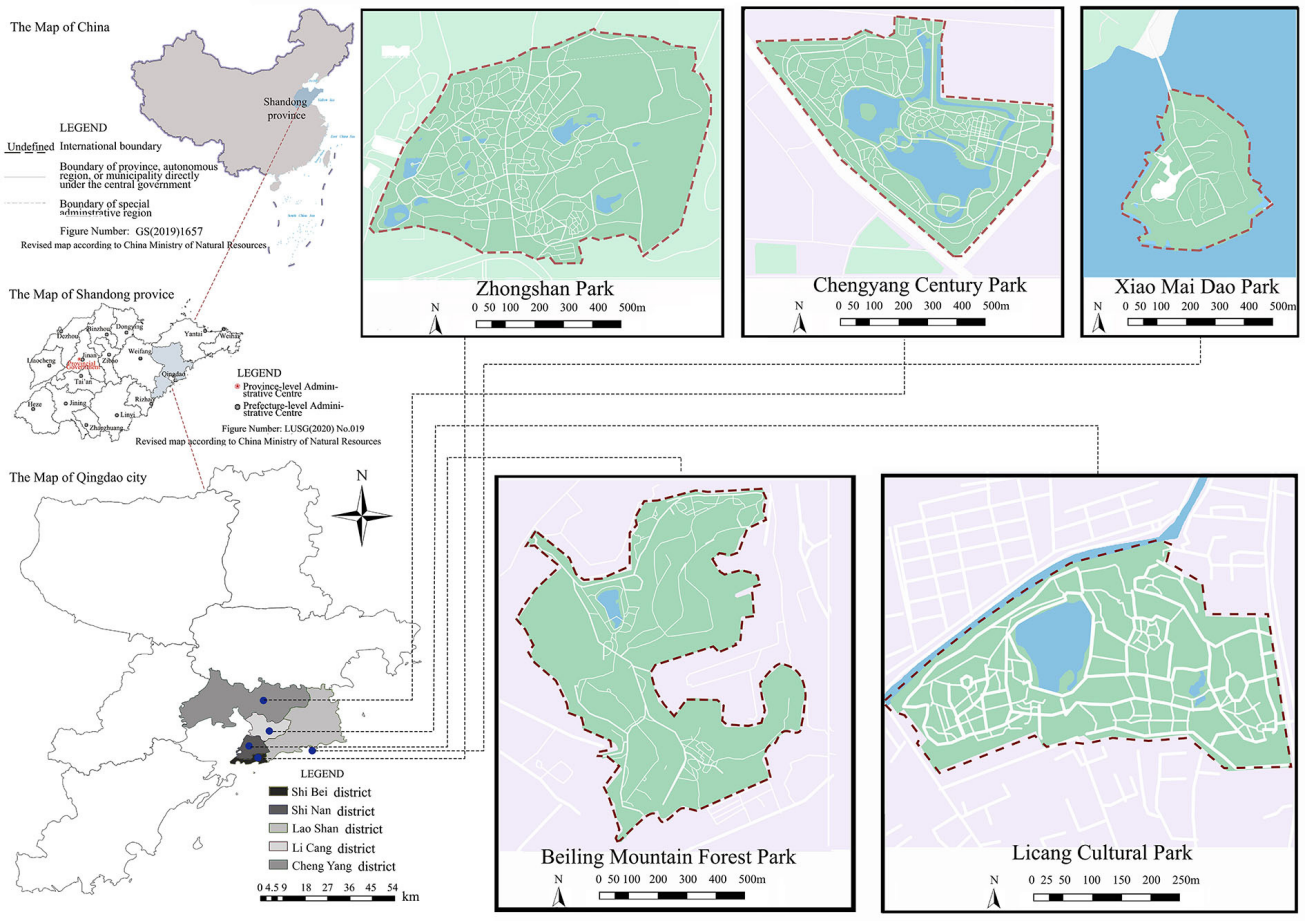
Study areas.

Qingdao, a prefecture-level city under the jurisdiction of Shandong Province, China, is an important coastal tourism center city and international port city. Up to now, Qingdao has 7 municipal districts and 3 county-level cities. According to the population statistics of administrative divisions released by the Ministry of Civil Affairs, PRC in 2019, Shinan District, Shibei District, Licang District, Chengyang District, and Laoshan District in Qingdao have higher population density, which is expected to continue to rise in the future. Therefore, urban parks in these five regions were selected for preliminary investigation, as shown in Figure 1. After excluding urban parks with an area of <10 ha, the current situations of urban parks were compared through network survey and field survey. At last, five parks of different areas and types were selected as the survey objects: Zhongshan Park, Chengyang Century Park, Xiao Mai Dao Park, Beiling Mountain Park, and Licang Cultural Park. These five parks have excellent landscape resources, with a greening rate of over 70%. They can attract many people to gather during rest days and are easily favored by youth, and the population is relatively stable. The specific features of the park are shown in Table 1.

**Table 1:** Description of the study area.

**Park name**	**Location**	**Area (ha)**	**Green rate(%)**	**Types**	**Park pictures**
Zhongshan Park	Shinan district	75	83	Comprehensive Park	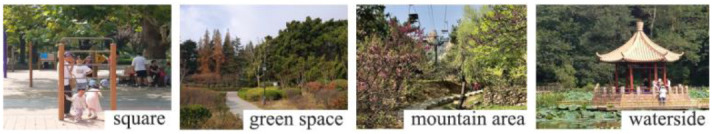
Chengyang Century Park	Chengyang district	43	89.5	Science education theme park	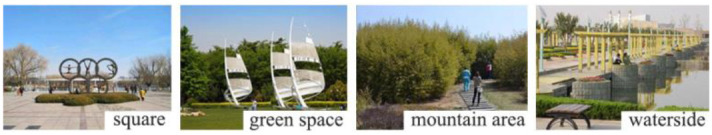
Xiao Mai Dao Park	Laoshan district	12.2	71.1	Coastal park	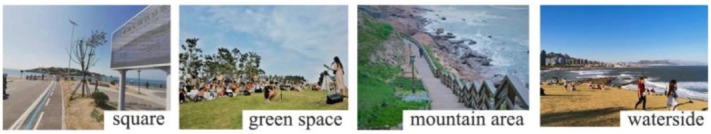
Licang Cultural Park	Licang district	17.46	81.4	Cultural theme park	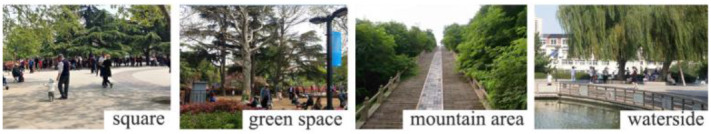
Beiling Mountain Forest Park	Shibei district	46.54	86	Forest park	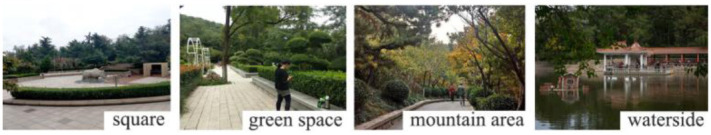

### Questionnaire survey

This research adopted the form of a semi-structured questionnaire (29–32). The main contents to be filled in include the following: (1) Sociological characteristics of the samples and spatial type of landscape used, including gender, education level, marital status, place of residence, and usage of urban parks. (2) Satisfaction of park landscape variables. According to previous research (33–37), we initially selected 15 variables that might affect youth's use of urban parks and conducted a correlation analysis of variables to avoid multicollinearity. Then, we screened after a preliminary survey in Zhongshan Park and 9 variables were finally determined, including waterscape, greenery landscapes, architectural landscapes, accessibility, facility quality, pavement quality, color perceptions, sound perceptions, and cultural perceptions. The options of the questionnaire are in the form of a five-level scale, which includes very satisfied, relatively satisfied, moderately satisfied, relatively dissatisfied, and very dissatisfied. The scores in order are 6, 3, 0, −3, and —, respectively. (3) Usage behavior and health perception benefits. Usage behavior is based on the data collected in the early stage and the usage behavior of youth in “Zhongshan Park,” the results can be classified into seven usage behaviors and four activity attributes (Supplementary table S1). The health perception benefits were provided by respondents. The most common responses and mention times were counted. Health perception benefits were mainly divided into the following three parts: physical health perception, mental health perception, and social health perception.

### Questionnaire reliability test

Cronbach's reliability test value for the questionnaire in this study is 0.758 (above 0.7), which indicates better internal consistency and a higher frequency of usage. The overall KMO of the questionnaire is 0.818, which is > 0.5, indicating that the study has sufficient samples and is suitable for factor analysis. The statistical significance of the Bartlett sphericity test is < 0.01%, indicating that the validity test can be performed.

### Research methods

The survey activity was conducted on weekends (8:00 am to 04:00 pm) when the weather was clear from October to November. The survey sites were distributed in different landscape spaces of the parks (square, green space, mountain area, and waterside). Three researchers (JY, TM, and ZY) used randomized questionnaires and in-depth conversations with youth to understand the interviewees' use of the target parks, such as activity requirements, park improvement suggestions, and how the parks could help their health, with a single visit taking approximately 20 min. A total of 500 questionnaires were distributed, of which 464 were valid, with an effective rate of 92.8%.

### Data processing

Behavior observation, communication interview, expert interview, photographic recording, and other methods were used to collect information. SPSS 24.0 software was applied for correlation analysis of data.

## Results

### Characteristics of the research subjects

The characteristics of youths aged 18–35 years who participated in the study were shown in Supplementary table S2. Approximately 80% of the respondents had advanced degrees. Single youth who used urban parks accounted for 53.45%, with Xiao Mai Dao Park having the largest number of single people. Approximately half of the users reported a monthly income of more than 7,000 CNY/month. The clear majority of the respondents spent < 2 h in the park, and 42.67% of respondents spent 0.5–1 h per visit in the park. Nearly, half of the respondents used the park once or twice a week (41.81%). According to the field research, it was found that the number of youths interviewed was the largest in green space (40.30%), and least in squares (17.24%).

### Satisfaction of park landscape variables

**Figure 2:**
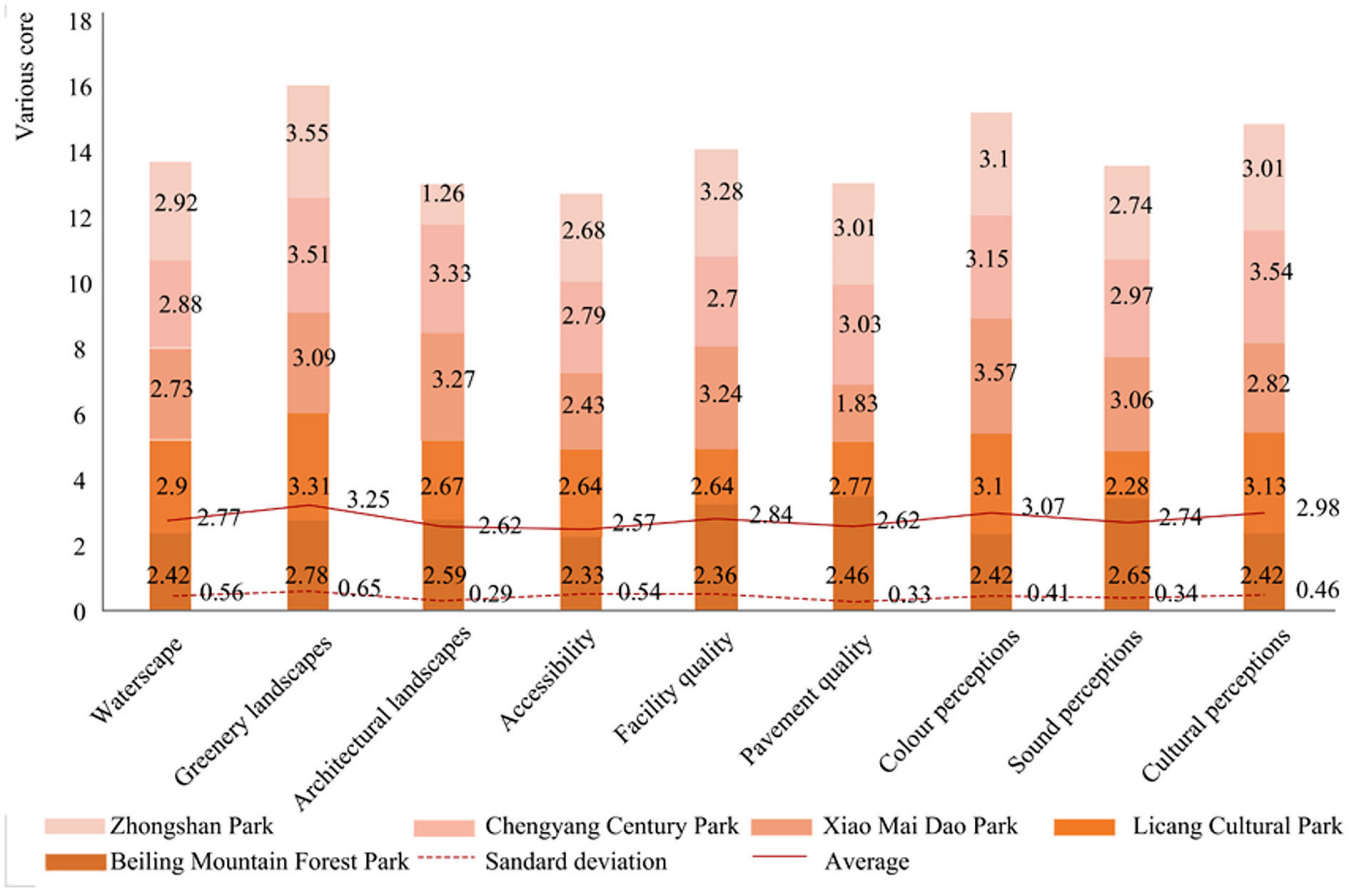
Satisfaction of park landscape variables.

According to the statistical results of interviewees' satisfaction evaluation of urban parks, the scores and averages of the nine variables for five parks were calculated (Figure 2). Compared with other variables, the greenery landscape scored the highest in youth's evaluation (3.25 ± 0.65) was of higher value, followed by color perception (3.07 ± 0.41) and cultural perception (2.98 ± 0.46). Accessibility got the lowest score (2.57 ± 0.54).

### Usage behavior of youth

**Figure 3:**
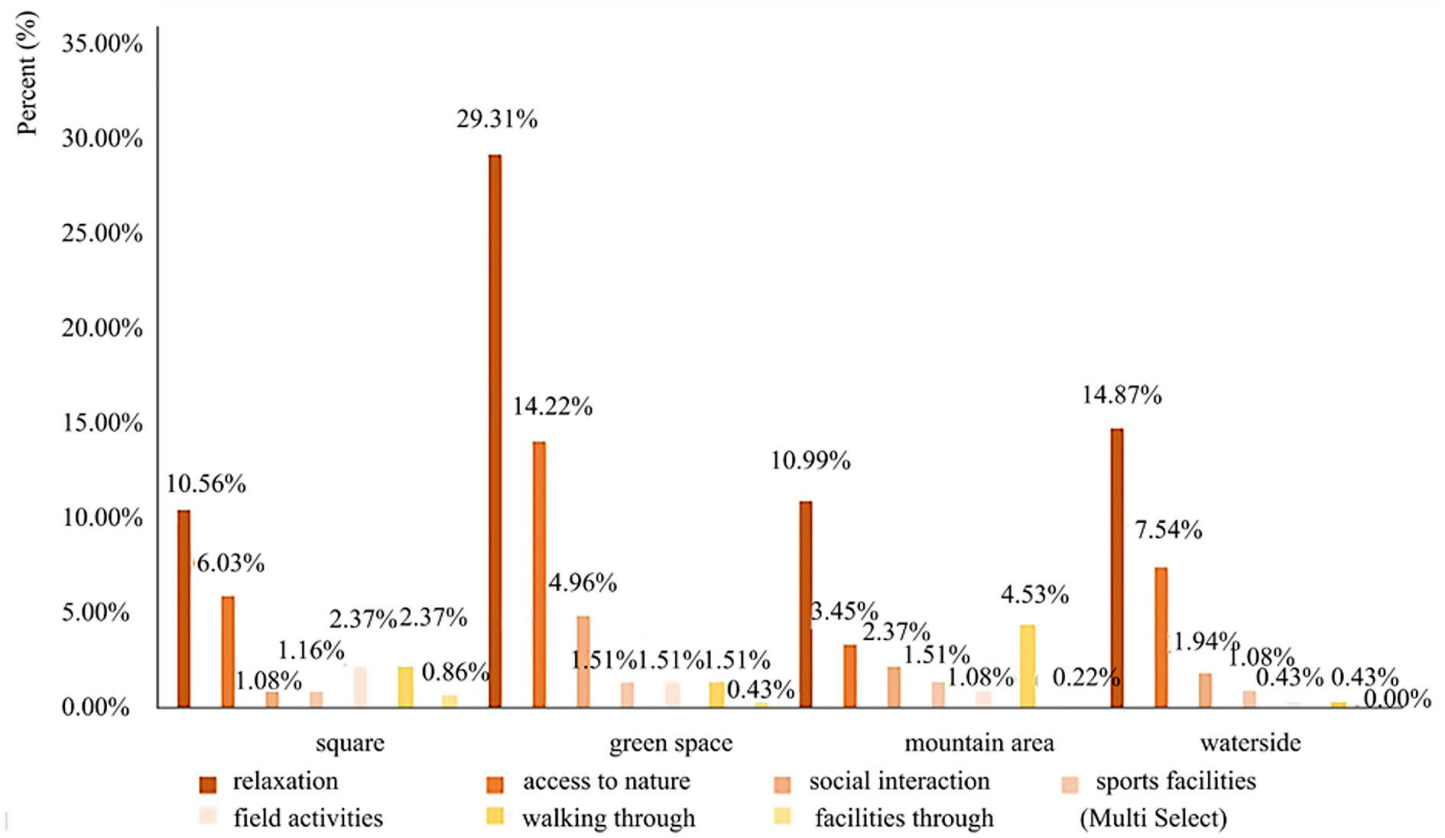
Use behavior in four spatial types of urban parks.

According to the questionnaire survey and field observation, there are 7 main types of youth's usage behavior in urban parks as shown in Figure 3. More youths chose to “relaxation” (65.73%) and “get close to nature” (31.24%) while fewer of them chose to “facilities through” (1.51%).

### Health benefit perception

**Figure 4:**
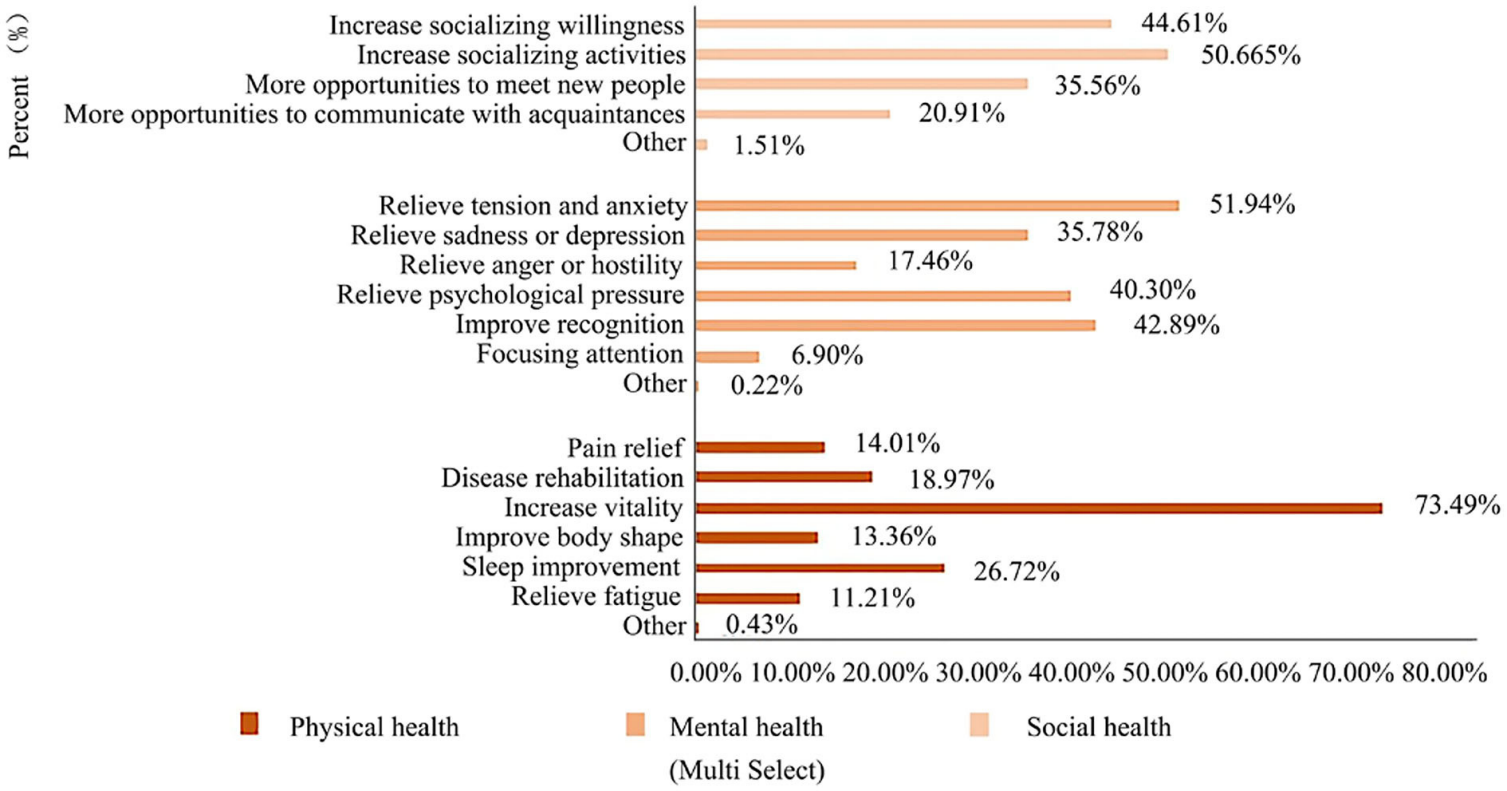
Health perception of youth to urban parks.

The results of the questionnaire showed that youth overall believed that urban park uses improved their health, with the most significant benefits in mental health (Figure 4). We observed that in terms of physical health (38), 73.49% of youths thought that using urban parks could increase vitality, and 11.21% of youths thought it could relieve fatigue. In addition, in terms of mental health (39), more respondents believed that it could relieve tension and anxiety (51.94%) and improve recognition (42.89%), and only 6.90% of youths thought it could improve attention. At last, 50.65% of the participants believed that using urban parks would increase socializing activities and contribute to social health (40–42).

### Correlation analysis

The data of park users were analyzed from three aspects: park landscape variables, usage behavior, and health benefit perception of urban parks. Besides, three models of “landscape variables–usage behavior,” “landscape variables–health perception” and “usage behavior–health perception” were established. These models were used to further explore the influence mechanism on park users.

#### Landscape variables and usage behavior

The variables of urban park landscape and youth use behaviors were analyzed by unary linear regression (Table 2). As more interviewees chose relaxation and assess to nature in parks, taking these as the focus of the analysis, there was an extremely significant (*p* < 0.01) positive correlation between the relaxation behaviors of park users with waterscape (*p* = 0.002) and greenery landscapes (*p* = 0.001) of the urban parks. In addition, it also had a significant (*p* < 0.05) correlation with accessibility (*p* = 0.012), color (*p* = 0.018), and cultural perceptions (*p* = 0.015). Park users' behavior of assess to nature significantly correlated (*p* < 0.05) with the color perceptions (*p* = 0.020) and cultural perceptions (*p* = 0.006) of the urban parks. While there was a significant negative correlation between park users' facilities through behaviors and greenery landscapes (*p* = −0.029). This might be due to behavioral inhibition of the use of skateboards, bicycles, and other facilities in green spaces and areas with rich plant community structures.

**Table 2:** Unary linear regression model of “landscape variables—usage behavior.”

	**Waterscape**	**Greenery landscapes**	**Architectural landscapes**	**Accessibility**	**Facility quality**	**Pavement quality**	**Color perceptions**	**Sound perceptions**	**Cultural perceptions**
Relaxation	0.002**	0.001**	0.232	0.012*	0.110	0.346	0.018*	0.080	0.015*
Access to nature	0.241	0.349	0.166	0.209	0.104	0.068	0.020*	0.427	0.006**
Social interaction	0.022*	0.066	0.078	0.074	0.033*	0.274	0.140	0.289	0.208
Sports facilities	0.289	0.464	0.449	0.252	0.484	0.137	0.200	0.166	0.401
Field activities	0.110	0.073	0.312	0.336	0.327	0.396	0.187	0.471	0.257
Walking through	0.212	0.221	0.431	0.336	0.046*	0.223	0.157	0.022*	0.023*
Facilities through	0.104	-0.029*	0.073	0.392	0.032*	0.048*	0.022*	0.620	0.287

^*^Significant at p < 0.05;

^**^significant at p < 0.01.

Bold values are significant differences.

The aforementioned research results indicated that urban parks, as larger green spaces in cities, tend to have better accessibility, leisure facilities, and walking paths, thus, providing more opportunities for various activities. Therefore, different usage behaviors of the residents in the park had different needs for landscape variables. It was shown that youths paid more attention to the quality of the natural landscapes in the urban parks when they were engaged in static behavior. In the terms of dynamic behaviors, participants had higher requirements for facility quality, sound perception, and cultural perception. The “comfortable facilities” and “cultural places” in the parks were more attractive to youths. Moreover, most youths would consider corridors, viewing halls, monuments and landscape places, etc., as places to stay first and foremost.

#### Landscape variables and health perception

According to the result of satisfaction of park landscape variables in Figure 2, the youths surveyed were more satisfied with the greenery landscapes, color perceptions, and cultural perceptions. In Table 3, both greenery landscapes and colour perceptions had significant correlation with disease rehabilitation (*p* < 0.05), relieve tension and anxiety (*p* < 0.01), and increase socializing activities (*p* < 0.05). However, the cultural perceptions of the park were not related to the health perception. Moreover, the improved recognition was highly correlated (*p* = 0.014) with green landscape. In addition, the water landscape also had a significant impact on youth' health perception, which was significantly correlated with disease recovery (*p* = 0.002), increased social activities (*p* = 0.001), and more opportunities to meet new people (*p* = 0.016).

**Table 3:** Unary linear regression model of “landscape variables—health perception”.

		**Waterscape**	**Greenery landscapes**	**Architectural landscapes**	**Accessibility**	**Facility quality**	**Pavement quality**	**Color perceptions**	**Sound perceptions**	**Cultural perceptions**
Physical health	Pain relief	0.607	0.464	0.633	0.076	0.431	0.869	0.270	0.626	0.674
	Disease rehabilitation	0.002**	0.015*	0.499	0.012*	0.708	0.546	0.014*	0.885	0.952
	Increase vitality	0.371	0.683	0.222	0.505	0.042*	0.184	0.661	0.082	0.168
	Improve body shape	0.677	0.147	0.312	0.061	0.979	0.242	0.196	0.101	0.297
	Sleep improvement	0.820	0.059	0.283	0.177	0.016*	0.672	0.350	0.184	0.129
	Relieve fatigue	0.051	0.462	0.312	0.462	0.462	0.460	0.462	0.645	0.461
Mental health	Relieve tension and anxiety	0.062	0.001**	0.048*	0.073	0.481	0.589	0.001**	0.437	0.086
	Relieve sadness or depression	0.771	0.653	0.089	0.262	0.240	0.095	0.380	0.678	0.903
	Relieve anger or hostility	0.819	0.484	0.787	0.064	0.642	0.490	0.143	0.760	0.679
	Relieve psychological pressure	0.8 72	0.767	0.261	0.441	0.882	0.277	0.615	0.234	0.295
	Improve recognition	0.149	0.014*	0.415	0.789	0.876	0.811	0.397	0.022*	0.882
	Focusing attention	0.898	0.883	0.692	0.132	0.876	0.826	0.998	0.937	0.634
Social health	Increase socializing willingness	0.305	0.194	0.939	0.707	0.703	0.506	0.052	0.112	0.858
	Increase socializing activities	0.001**	0.001**	0.007**	0.721	0.617	0.042*	0.832	0.054	0.522
	More opportunities to meet new people	0.016*	0.062	0.172	0.966	0.345	0.756	0.426	0.123	0.318
	More opportunities to communicate with acquaintances	0.306	0.854	0.341	0.187	0.354	0.207	0.264	0.958	0.524

^*^Significant at p < 0.05;

^**^significant at p < 0.01.

Bold values are significant differences.

According to our results, higher quality of waterscapes and greenery landscapes contributed to human disease rehabilitation, relieve tension and anxiety, and other social health. The environmental improvement brought about by the presence of green plants and water made youth more relaxed and comfortable, helping them improve their senses, and overall physical health, as well as providing a good space for socializing. On the contrary, the higher quality of architectural landscapes and richer cultural perceptions were effective in increasing socializing activities and reducing youth's anxiety and tension. The results also indicated that people at different health levels had different needs for park landscapes, and the users who had greater physical stress wanted park landscapes with more naturality (43). Moreover, some of the potential barriers in the park might also influence usage behaviors. For example, if the facilities in a park were not convenient and perfect, their functions would be reduced and would affect the usage of urban parks.

#### Usage behavior and health perception

Through the analysis of the user behavior and health perception of interviewees (Table 4), it was clear that there was no significant correlation between passing behavior (walking through and facilities through) and health perception. For the static behavior, relaxation was highly associated with focusing attention (*p* = 0.001), followed by relieving tension and anxiety (*p* = 0.014) and more opportunities to communicate with acquaintances (*p* = 0.016). Accessing to nature had an extremely significant (*p* <0.01) with sleep improvement and relieving tension and anxiety. Moreover, social interaction had significant correlations with relieving tension and anxiety (*p* = 0.023), improving recognition (*p* = 0.001), increasing socializing willingness (*p* = 0.007), increasing socializing activities (*p* = 0.017), and more opportunities to meet new people (*p* = 0.004). As the aforementioned results indicated that, with the exclusion of passing behavior, namely, static behavior, dynamic behavior in urban parks could greatly reduce the health problems of youth. For example, surroundings filled with plants and animals, popular science education of plants, running, playground entertainment, and venue activities could immensely improve the physical and mental health of youth. Social health effects could be improved by increasing socializing willingness through social communication and sports facilities, such as chat-gathering, picnic, fitness equipment activities, and leisure facilities activities.

**Table 4:** Unary linear regression model of “usage behavior— health perception.”

		**Relaxation**	**Access to nature**	**Social interaction**	**Sports facilities**	**Field activities**	**Walking through**	**Facilities through**
Physical health	Pain relief	0.064	0.093	0.299	0.539	0.737	0.300	0.355
	Disease rehabilitation	0.207	0.917	0.986	0.348	0.154	0.072	0.158
	Increase vitality	0.254	0.244	0.841	0.555	0.199	0.555	0.449
	Improve body shape	0.230	0.349	0.382	0.133	0.388	0.697	0.445
	Sleep improvement	0.471	0.009**	0.969	0.111	0.353	0.898	0.439
	Relieve fatigue	0.632	0.251	0.464	0.733	0.037*	0.306	0.446
Mental health	Relieve tension and anxiety	0.014*	0.008**	0.023*	0.805	0.265	0.478	0.193
	Relieve sadness or depression	0.799	0.099	0.885	0.254	0.020*	0.571	0.130
	Relieve anger or hostility	0.427	0.723	0.082	0.533	0.001**	0.612	0.539
	Relieve psychological pressure	0.755	0.115	0.081	0.801	0.468	0.553	0.880
	Improve recognition	0.674	0.598	0.001**	0.464	0.198	0.894	0.591
	Focusing attention	0.001**	0.845	0.184	0.053	0.180	0.885	0.462
Social health	Increase socializing willingness	0.426	0.072	0.007**	0.003**	0.496	0.534	0.534
	Increase socializing activities	0.441	0.119	0.017*	0.119	0.246	0.464	0.469
	More opportunities to meet new people	0.292	0.469	0.115	0.534	0.278	0.469	0.278
	More opportunities to communicate with acquaintances	0.016*	0.278	0.004**	0.465	0.254	0.172	0.462

^*^Significant at p < 0.05;

^**^significant at p < 0.01.

Bold values are significant differences.

## Discussion

Urban parks provide high-quality landscape and leisure places for surrounding areas, and they are helpful for the vitality and innovation functions of urban and promote the inheritance of local culture. Meanwhile, their internal facilities and resources also help build popularity, youth can be attracted to the different physical activities and improve their health benefits.

### Different usage behaviors need to be considered

Through the behavioral observation, questionnaire and interview, the differences in using motivations of interviewees can be attributed to different socioeconomic conditions, lifestyle preferences, or personal reasons. Meanwhile, these results proved that different spatial preferences can largely explain the usage behaviors of youth. It has shown that youths are more likely to relax, have access to nature, and engage in social interaction in green and waterspaces. They are more likely to engage in playing, fitness, running, other leisure, and field activities in square space. However, the results also reflect some problems, youth in urban parks generally have fewer types of activities, so we suggest that more diversified types of the usage behavior should be considered. For example, provide some challenging and interesting facilities in the open space. Increase youth's willingness to visit and satisfaction by improving the accessibility and pavement quality of parks, adding outdoor education courses, plant crafts, and horticultural displays.

### Urban park landscape is closely related to usage behavior

The urban park landscape plays an important role in promoting and inhibiting the youth's usage behavior. Therefore, factors that show a significant correlation between park landscapes and usage behavior should be incorporated into the urban park design. The static behaviors of youth in parks, such as relaxation and social interactions, are mostly related to natural landscapes and accessibility in the urban parks The environmental features that impede the motivation of “facilities through” were copious shrubs and terrain changes. Thus, landscape designers need to pay more attention to elements, such as plants, water, and microclimate building. For example, a combination of open water, densely trees, open lawns, and hilly terrain to create more relaxing, entertaining activities. Circular trails are planned around the waterscape to create opportunities for walking and running activities. Though clearing obstructions line of sight, visible landmarks of different shapes and scales are placed outside the space to increase the visual attraction elements in the activity space, enhance the interaction between landscape and users, and enrich the effect of the activity experience.

### Strengthening waterscape and green landscape construction is an important way to improve health perception

According to the results, youths are generally aware of the positive effects of urban parks on their health. In urban parks, waterscape and greenery landscapes are the most significant and stable factors that can influence youth's usage behavior and health benefit perception. In previous studies, water is considered to be an important landscape variable to relieve visual fatigue and to increase sensory comfort. After experiencing waterscape viewing, young students' attention and negative emotions can be recovered significantly, and their cognitive function can be improved in the short term (44–46), which suggests the importance of creating high-quality waterscape and contributing to the development of a healthy landscape within the park. Combined with the observation results, the visuospatial near the water is more attractive to youth, which is conducive to static behavior. In the design of waterscape, so different structures can be used to create morphometric variations in the spatial scales, and rest platforms can also be used to attract more youths. But these structures have certain shading and resting effect, designers should consider the positioning problem to meet the needs of users to have shade in summer and sun in winter.

At the same time, landscape designers are suggested to include variable water features and plants with typical health functions. For example, plants such as ginkgo and cedar can be used for their beneficial volatiles and ecological benefits, such as sterilization and dust retention (47, 48). The physical health of youth can be promoted. In the survey, park users have given many valuable suggestions for “urban quality improvement.” Their suggestions mainly focus on “plant selection” and “growth management.” In the terms of plant selection, the mortality rates of famous flowers and trees transplanted in the park are higher, and there are not enough plants corresponding to the environment. Therefore, landscape architects are advised to use more indigenous plants and to reduce unnecessary transplanting of valuable species. These practices would in turn save resources and prevent energy consumption. Landscape designers are also advised to avoid plants that attract mosquitoes. In addition to ensuring the greening rate, emphasis should be placed on the arrangement of plant community structure. Vertical greening and shading trees should be added, as well as trees that bloom in all seasons and that have a pleasant scent. Finally, the edible and educational functions of the plants should be considered appropriately. In relation to plant management, park staff are advised to trim plants regularly and to carry out pest control.

### Consider static behavior in urban parks as an important way to improve health

Previous studies have shown that exercise is important in preventing non-communicable chronic diseases (49). It is especially important as a means of preventing mental health problems, such as chronic physical diseases (cervical spondylosis, heart disease, and obesity), depression and anxiety caused by stressful, fast-paced life and reduced exercise (50). Many scholars have also noted that outdoor dynamic activities are good for human health (51).

However, this study has found that non-dynamic behaviors also help youth's physical and mental health, specifically, some static behaviors in urban parks can improve their health perception. Youth's static usage behaviors, such as getting close to nature and participating in social interactions, are more closely related to their physical, mental, and social health. The aforementioned results suggest that park design should cater more to the needs of youth for static use. For example, some rest facilities beside green landscape can help youth to relax and decompress while supporting social interaction, improve the quality of leisure facilities, and increase the recreation space under the tree. The safety and health of internal space should be a priority, to promote the construction of healthy landscapes in urban parks and improve the health of youth.

## Limitations and future research

There are some limitations that should be considered. The study was conducted only in autumn, with a genial climate, generalization of our findings to the other seasons should be performed with caution. Furthermore, previous research by Wei, D. et al. (52) examines human thermal comfort in different landscapes of an urban park. Outdoor thermal comfort effects should also be taken into account in the experiment to obtain richer conclusions about youth use behavior and health perceptions in urban parks. It is widely accepted that the environment and health enhancement are inseparable, yet the mechanisms through which people perceive the environment or in different ways are complex and multisensory, so identifying the reasons for the differences between them remains to be further investigated.

## Conclusion

Youth as an important group of park users, often have different needs for the health benefits of urban parks. Field observation and questionnaires have been used in our study to explore the potential relationship between youth's satisfaction with park landscape variables, their usage behaviors and health benefit perceptions. Our study shows that:(1) most youths in urban parks prefer to engage in static activities, especially in greenspace; (2) youth believe that urban parks could help health recovery mainly in increasing vitality, relieving tension and anxiety, and increasing socializing activities; (3) the main landscape variables affecting usage behaviors in the sample urban parks are waterscape, greenery landscapes, color, and cultural perceptions; (4) well-maintained waterscape, greenery landscape, and facility quality are more effective to promote health; (5) accessing to nature, relaxation, and social interaction are more conducive to relieve tension and anxiety. Social interaction helps to improve social health effectively.

Our study could provide useful references for the construction of urban parks in the future, to promote the health of residents, and to ensure their sustainability, longevity, and frequent use. Therefore, it is recommended that urban planners should improve the physical environment, aesthetic aspects of urban parks and pay attention to landscape variables in urban parks, including greenery landscape, waterscape, color perceptions, and cultural perceptions, which may improve the health perception of youth. In conclusion, we hope that urban planners and landscape architects will make timely adjustments based on our findings in order to provide a satisfying experience for all the visitors, effectively improve their willingness to use the park and thus help to promote their health.

## Data availability statement

The original contributions presented in the study are included in the article/Supplementary material, further inquiries can be directed to the corresponding author.

## Ethics statement

Our study has been approved by Qingdao University of Technology and we confirm that the participants provided their written informed consent to participate in this study.

## Author contributions

WY: project management and funding acquisition. JY: conceptualization, data organization, data processing, and analysis. WY and JY: writing and methodology. YZ and WY: paper revision. JY, TM, and ZM: data collection. All authors contributed significantly to the study and approved the submitted version.

## Funding

This work was funded by the National Natural Science Foundation of China (No. 32001367), Open Fund of Innovation Institute for Sustainable Maritime Architecture Research and Technology (iSMART), Qingdao University of Technology (No. C2020-037), and Science and Technology Innovation Project for College students (KJCXXM084).

## Conflict of interest

The authors declare that the research was conducted in the absence of any commercial or financial relationships that could be construed as a potential conflict of interest.

## Publisher's note

All claims expressed in this article are solely those of the authors and do not necessarily represent those of their affiliated organizations, or those of the publisher, the editors and the reviewers. Any product that may be evaluated in this article, or claim that may be made by its manufacturer, is not guaranteed or endorsed by the publisher.
