# Supplementary material for: Usage behavior and health benefit perception of youth in urban parks: A case study from Qingdao, China
Source: Front Public Health. 2022 Aug 3;10:923671. doi: 10.3389/fpubh.2022.923671 (PMC9381962; doi:10.3389/fpubh.2022.923671)
Supplement: Supplementary file 1 [file Data_Sheet_1.pdf]

SUPPLEMENTARY MATERIAL

Tab.S1 Use behavior of young people in urban parks

Activity attribute	Usage behavior	Specific activity	Data source
Namely static behavior	relaxation	reading, meditation, playing, singing, drinking, …	questionnaire survey, field observation
	access to nature	ornamental plants, ornamental animals, plant science education, taking photos, …	questionnaire survey, field observation
	social interaction	chatting, meeting, playing cards and chess, picnics, …	questionnaire survey, field observation
Dynamic behavior	sports facilities	fitness equipment activities, amusement facilities activities, leisure facilities activities, …	questionnaire survey, field observation
	field activities	running, dancing, sword dancing, martial arts, playground entertainment, …	questionnaire survey, field observation
Passing behavior	walking through	walking, walk a pet, climb a mountain, take the kids, …	questionnaire survey, field observation
	facilities through	skateboarding, scooter riding, tramway, boating, …	questionnaire survey, field observation
Other behavior	/	merchandising, plant research, ...	field observation

Tab.S2 Sociological characteristics of the sample.

Variable	Description	Zhongshan Park	Chengyang Century Park	Xiao Mai Dao Park	Licang Cultural Park	Beiling Mountain Forest Park	Percentage (%)
Effective Questionnaire	Recovery rate (%)	100	100	100	92	72	
Gender	Male	55	47	48	53	35	51.29
	Female	45	53	52	39	37	48.71
Education	Primary / junior high school	7	8	2	12	5	7.33
	Technical secondary school / senior high school	14	10	8	20	7	12.72
	University / associate degree	58	57	58	39	34	53.02
	Bachelor / bachelor above degree	21	25	32	21	26	26.94
Marriage	Single	67	48	88	27	18	53.45
	Married	32	50	8	62	49	43.32
	Divorced	1	2	3	2	3	2.37
	Widowed	0	0	1	1	2	0.86
Residence	In Qingdao city	81	95	71	91	72	88.36
	Outside Qingdao city	19	5	29	1	0	11.64
Monthly income	≤ 800 CNY	29	22	24	17	12	22.41
	800 ~ 3,499 CNY	8	22	20	15	10	16.16
	3,500 ~ 6,999 CNY	6	10	9	30	20	16.16
	≥ 7,000 CNY	57	46	47	30	30	45.26
Duration of each use park	< 0.5 h	8	7	6	12	6	8.41
	0.5 ~ 1 h	43	48	46	37	24	42.67
	1 ~ 2 h	34	39	40	38	34	39.87
	> 2 h	15	6	8	5	8	9.05
Frequency of weekly use park	< 1 time	59	38	42	27	21	40.30
	1 ~ 2 times	34	35	51	40	34	41.81
	3 ~ 4 times	4	18	1	22	9	11.64
	> 5 times	3	9	6	3	8	6.25
Spatial type of landscape used	Square	24	15	17	14	10	17.24
	Green space	31	49	37	42	28	40.30
	Mountain area	31	4	25	22	25	23.06
	Waterside	14	32	21	14	9	19.40
